# Bt Toxin Modification for Enhanced Efficacy

**DOI:** 10.3390/toxins6103005

**Published:** 2014-10-22

**Authors:** Benjamin R. Deist, Michael A. Rausch, Maria Teresa Fernandez-Luna, Michael J. Adang, Bryony C. Bonning

**Affiliations:** 1 Department of Entomology, Iowa State University, Ames, IA 50011, USA; E-Mails: bdeist@iastate.edu (B.R.D.); mrausch@iastate.edu (M.A.R.); tfdzluna@iastate.edu (M.T.F.-L.); 2 Departments of Entomology, University of Georgia, Athens, GA 30602, USA; E-Mail: adang@uga.edu; 3 Department of Biochemistry and Molecular Biology, University of Georgia, Athens, GA 30602, USA

**Keywords:** *Bacillus thuringiensis*, Bt, toxins, genetic modification, crop protection, pest management, truncation, phage display, site-directed mutagenesis

## Abstract

Insect-specific toxins derived from *Bacillus thuringiensis* (Bt) provide a valuable resource for pest suppression. Here we review the different strategies that have been employed to enhance toxicity against specific target species including those that have evolved resistance to Bt, or to modify the host range of Bt crystal (Cry) and cytolytic (Cyt) toxins. These strategies include toxin truncation, modification of protease cleavage sites, domain swapping, site-directed mutagenesis, peptide addition, and phage display screens for mutated toxins with enhanced activity. Toxin optimization provides a useful approach to extend the utility of these proteins for suppression of pests that exhibit low susceptibility to native Bt toxins, and to overcome field resistance.

## 1. Introduction

The ubiquitous Gram-positive spore-forming bacterium *Bacillus thuringiensis* (Bt) provides a valuable resource due to its ability to synthesize crystal parasporal inclusions during sporulation [1]. These crystals, which include insecticidal proteins called δ-endotoxins have been extensively used as biological insecticides against insect pests of commercial interest [1]. More than 175 million hectares were planted to transgenic crops expressing Bt toxins in 2013, highlighting the practical importance of these toxins for pest suppression [2].

Bt crystal toxins include the Cry proteins (crystal toxins) and Cyt proteins (cytolytic toxins) [3,4]. While the ETX/MTX-like and the Binary (Bin)-like toxins do have Cry designations, they belong to structurally different classes of Cry toxins; we refer the reader to an updated review on Cry toxins diversity (4). The Cry toxins are important virulence factors allowing for the development of the bacterium in dead or weakened insect larvae. The largest group of Cry toxins has three distinct structural domains; the so-called three-domain (3D) Cry toxins provide the primary focus for this review. The Cyt toxins have *in vitro* cytolytic activity, in addition to *in vivo* activity against various insects including mosquitos [5]. Cyt toxins which are active against certain Diptera, synergize the toxicity of Cry proteins against mosquitoes and delay the expression of resistance to the latter [6]. While most Bt crystal toxins are encoded by large plasmids, with expression under the control of sporulation-dependent promoters, there are also cryptic Cry toxins that are not expressed [1,7]. The high level of crystal protein expression is controlled by a variety of mechanisms occurring at the transcriptional, post-transcriptional, and post-translational levels [5].

The evolution of resistance by targeted pests is the main threat to the usefulness of Bt toxins. We outline Bt toxin mechanism of action to provide the framework for toxin modification to combat resistance, or to broaden host range.

## 2. Insecticidal Bt Proteins

### 2.1. Mode of Action of Bt Toxins

**Figure 1:**
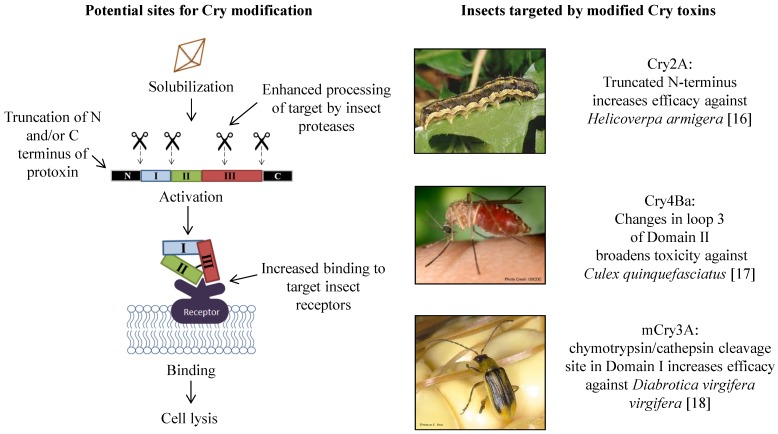
Schematic representation of the major steps involved in Cry toxicity and sites for modification to increase efficacy and/or broaden toxicity. Some of the insect species successfully targeted by modified toxins are shown at right [16,17,18].

The conserved structure of 3D Cry toxins, as well as results from a considerable amount of research, supports a conserved mode of Cry toxin action [5,8]. The first impact of Cry toxins on the insect is cessation of feeding due to paralysis of the gut and mouthparts [9]. In addition to gut paralysis, midgut cells swell leading to an ion imbalance and death [9]. The molecular events leading to Cry toxin-mediated insect death are controversial [8], but the accepted initial steps are as follows: The Bt Cry and Cyt proteins require solubilization in the insect midgut to produce protoxins that are typically about 130 kDa, 70 kDa or 27 kDa for Cyt. These in turn are proteolytically cleaved at the *C*-terminus and/or at the *N*-terminus by midgut proteases, generating the activated core toxin. The toxin then crosses the peritrophic matrix and binds to receptors in the apical membrane of the midgut cells, with receptor binding being an important determinant of toxin specificity. Toxin insertion into the epithelial membrane forms ion channels or pores, leading to lysis of the cells, damage to the midgut epithelial tissue, and death of the larva (Figure 1) [8,10,11].

### 2.2. Structure of Bt Toxins and Domain Function

Cry protoxins are typically 130 kDa or 70 kDa proteins [5] with the larger protoxins having an additional carboxyl region that is not required for toxicity but that is required for crystal formation [3,5]. When these crystal proteins are exposed to the alkaline environment of the gut of susceptible larvae, the crystals are solubilized and proteolytically processed at the *N*-terminus and/or the *C*-terminus by midgut proteases to yield the active protease-resistant toxin [3,5].

Based on their crystal structures, different Cry toxins (Cry1Aa, Cry1Ac, Cry2Aa, Cry3Aa, Cry3Bb, Cry4Aa, Cry4Ba and Cry8Ea1 [3,12,13]) have similar folding patterns with three distinct domains [3,5]. Domain I is a seven to eight α-helix bundle comprised of amphipathic helices surrounding the central hydrophobic helix α-5. Domain I is involved in binding and in pore formation [3,5,14]. Domain II consists of three antiparallel β-sheets with exposed loop regions involved in interaction with receptors, while domain III is a β-sandwich of two antiparallel β-sheets involved in receptor binding and possibly membrane insertion. In contrast, Cyt toxins consist of a single domain in which two outer layers of α-helix are wrapped around a β-sheet [5,15].

The role of domain I in membrane insertion and pore formation in the midgut epithelium of the target insect was suggested by the presence of its long, hydrophobic and amphipathic helices [14]. The Umbrella Model proposed by those authors depicts the hydrophobic helical hairpin α4 and α5 as inserting into the membrane and initiating pore formation, while the rest of domain I flattens out on the membrane surface in an umbrella-like molten globule state. Mutant Cry proteins having altered amino acid residues on the putative surface residues of domain I and within the α-helices supported the role of domain I in membrane binding, insertion and pore formation [19].

Domain II is implicated in protein-receptor interactions through the surface-exposed loops at the apices of the three β-sheets. Due to their similarities to immunoglobulin antigen-binding sites, the loops of domain II were suggested to participate in receptor binding. Site-directed mutagenesis and segment swapping analyses provided support for this hypothesis [5,14]. The β-sandwich structure of domain III is also suggested to function in receptor binding; evidence for this role in toxin action comes from domain III swap experiments discussed below, and in the case of Cry1Ac, the presence of a “pocket” in domain III that binds *N*-acetyl galactosamine moieties on protein receptors [20,21]. Domain III is also implicated in maintaining the structural integrity of the toxin molecule by protecting it from proteolysis within the gut of the target organism [3,14].

The Cyt toxins characterized to date are also protoxins that undergo *N*- and *C*-terminal processing. The predominantly β-sheet core structure of Cyt2A suggests a mechanism of action through a β-barrel-pore [15]. It contains three strands long enough to span the hydrophobic lipid membrane, forming a hydrophobic sheet [15,22]. In addition to forming pores, Cyt toxins may exert their effects via a general, detergent-like perturbation of the membrane [23]; the Cyt toxins were recently reviewed [24,25].

## 3. Truncated Toxins

Through a systematic analysis of Cry toxin deletions, the role of the *N*- and *C*-terminal domains in crystal formation and in toxin activity against different insects has been elucidated. Bt 3D crystal protoxins form crystalline inclusions that probably provide protection against proteolytic degradation by the host cell or external environment. Crystal solubilization in the gut of the target insect is required, however, for toxin action. This solubilization step occurs in many cases through a series of cleavages of the *C*-terminal half of the protoxin. The *C*-terminal half of the protoxin is cysteine-rich with disulfide bonds and salt bridges that allow for crystal formation [3,14].

The truncation of Bt toxins has proven to be a useful option for enhancing Bt toxin activity. The goal of this strategy is to circumvent the toxin activation step resulting in improved toxicity against target pests. Toxin truncation may also improve expression levels *in planta* and deletion analyses yield important information about the molecular biology and mode of action of these toxins.

### 3.1. Deletion Analyses: Localization of the Toxic Portion of the Protoxin

Cry protoxins are proteolytically activated to produce the mature active toxin. This activation step restricts the spectrum of toxicity of these toxins to insects with appropriate gut proteases, with activation of the Bt crystal protein being essential for toxin action [9]. Low susceptibility may be due to lack of proper proteolytic activation or over-digestion of toxin to inactive peptides, and thus a basic knowledge of the toxic portion is helpful for design of new toxins to control non-susceptible pests. To identify the minimal gene fragment that encodes the toxic peptide, deletions at the 5'- or the 3'-end of the Cry1Ab gene from Bt Berliner 1715 [26] and Bt *kurstaki* HD-1-Dipel [27] and Cry1Ac of Bt *kurstaki* HD-73 [28] protoxins of ~130 kDa (~60 kDa activated) have been made. Analysis of Bt strains expressing the toxin-sized proteins, indicated that the *N*-terminal half of the protoxin is sufficient for toxicity [26,27,28]. Approximately 400 amino acids can be removed from the *C*-terminus of the toxin without significant loss of larvicidal activity. Several Cry1A toxins are proteolytically cleaved at residue 28 at the *N*-terminus [29], followed by removal of α-helix 1 upon contact with the brush border membrane [30]. Notably, the minimal toxic fragment corresponds to the trypsin-resistant polypeptide [26,29,31]. The full-length (72 kDa) and truncated (61 kDa) forms of Cry11A were expressed in cells of the fall armyworm (*Spodoptera frugiperda*) and in larvae of the cabbage looper (*Trichoplusia ni*) by using a baculovirus expression vector. While the full-length protein was highly toxic to mosquito larvae, the truncated protein with a 9.6 kDa deletion at the *N*-terminus was non-toxic [32]. This result corroborates the requirement for most of the *N*-terminal region either directly or indirectly (*i.e.*, required for appropriate folding) for toxicity.

Crystallographic structural studies [33] were of tremendous value for prediction of functions of the various Cry domains. Based on structural predictions*,* deletion of 42 amino acid residues from the *N*-terminus of Cry2A domain I resulted in a 4- to 6-fold increase in toxicity against the Egyptian cotton leafworm (*Spodoptera littoralis*), cotton bollworm (*Helicoverpa armigera*), and the black cutworm (*Agrotis ipsilon*) [16]. It was speculated that the interaction of the *N*-terminal hydrophilic helix within the putative transmembrane domain I could interfere with toxin insertion into the membrane, which is required for toxicity [16].

The carboxy-terminal extensions of many Cry toxins mediate formation of bipyramidal crystals that are soluble at high pH under reducing conditions, typical of the lepidopteran midgut. Deletion of the Cry15Aa *C*-terminal sequence showed that this sequence is not required for activity against the codling moth (*Cydia pomonella*) [34]. In contrast, bipyramidal crystal formation precluded activity against the Colorado potato beetle (*Leptinotarsa decemlineata*). In this case, activity could be rescued by solubilization of the toxin [35].

Knowledge of the toxic portion of a given toxin is also useful for Cry gene shuffling. The Cry1Ia truncated protein lacking the *C*-terminus was used to generate mutated variants, by combining Cry gene shuffling with phage-display [36]. The screening of this library for variants that bind brush border membrane vesicles (BBMV) of the banana stem borer (*Telchin licus licus*) and for toxicity, revealed four variants with greater activity against this pest compared to the non-toxic wild type Cry1Ia. These variants provide promising candidate toxins for the future development of *T. l. licus* resistant transgenic plants.

The truncated Bt toxin gene without the protoxin *C*-terminus was used for expression of the toxin in the early stages of developing Bt transgenic plants. The Cry1A protoxin was toxic to transgenic tobacco plants [37] while the toxin truncated at the *C*-terminal proteolytic cleavage site that generates the mature toxin, was non-toxic to the plants [37]. The truncated toxin did confer resistance to the tobacco hornworm (*Manduca sexta*), however. Furthermore, expression levels in transgenic tobacco sufficient for insecticidal activity were only achieved with the truncated Cry1A, and not with the Cry1A protoxin [38].

The extensive use of Bt toxins has resulted in field-evolved resistance in some insect pests. Toxin modification for greater efficacy against resistant insects has been achieved with the genetically modified Cry1AbMod and Cry1AcMod toxins. Compared to Cry1Ab and Cry1Ac, both Cry1AbMod and Cry1AcMod lack 56 amino acids at the *N*-terminus, including the α-1 helix in domain I. Resistant insects with cadherin gene mutations such as pink bollworm (*Pectinophora gossypiella*) [39] were susceptible to Cry1AMod toxins, supporting the hypothesis that Cry1AbMod bypasses the toxin oligomerization step induced by binding to cadherin. Interestingly, these Cry1AMod toxins were also toxic to strains of the diamondback moth (*Plutella xylostella*) and European corn borer (*Ostrinia nubilalis*) which are resistant to the native toxins Cry1Ab and Cry1Ac, but in these examples resistance was not linked to mutations in cadherin [40]. Recently, Cry1AbMod toxin was reported to counter resistance due to low cadherin expression, but not low alkaline phosphatase expression [41].

### 3.2. Improved Expression of Truncated Toxins

In addition to enhanced *in planta* expression of the truncated toxin compared to expression of the protoxin, Hayakawa *et al.* [42] designed a gene encoding the truncated Cry4Aa with the GC content and codon preference of *E. coli*. These modifications resulted in significant improvements in expression levels in *E. coli*, with the recombinant toxin exhibiting the same toxicity against larvae of the common house mosquito (*Culex pipiens*) as the native Cry4Aa.

Park *et al.* [43] obtained inclusions of truncated Cry1C (Cry1C-t) by combining genetic elements from other endotoxin genes and operons that enhance Cry protein synthesis. Increased levels of Cry1C-t synthesis were achieved by using Cyt1A promoters to drive expression of the *N*-terminal half of Cry1C, shown to be toxic to beet armyworm larvae (*Spodoptera exigua*) when expressed in the HD1 isolate of *B. thuringiensis* subsp. *kurstaki* [43].

## 4. Cleavage Site Modifications

Processing of the Cry protoxin into its active form is essential for toxin activity. Processing is mediated by insect proteases that cleave the protoxin polypeptide at specific sequences. Refer to [44] for a detailed review of the role of proteases in Cry toxin action. Processing of protoxins has been well described in the 3D group of Cry toxins. Cleavage of the 3D Cry toxins results in the removal of *N*-terminal peptides, and occasionally *C*-terminal peptides in larger protoxins, producing the active toxin (Cry3) or a toxin intermediate (Cry1, Cry4, Cry11). For the latter, activation is completed by proteolysis at additional intramolecular sites to produce the active toxin fragments [5,45,46,47]. Proteolytic cleavage at these sites is required for subsequent events involved in toxicity [18,48,49], and may be exploited in the design and construction of modified toxins [18,50]. Proteolytic cleavage that results in toxicity is referred to as processing or activation. This section describes evidence supporting proteolytic cleavage as an activation step in 3D Cry toxins and how this process can be utilized for toxin modification.

### 4.1. Toxicity and Protoxin Processing

One of the initial observations that suggested proteolytic processing as a toxin activation step is that feeding a susceptible insect species on protoxin or on activated toxin resulted in similar levels of mortality, and that toxicity could be attenuated when protoxin was fed in the presence of protease inhibitors [18,48,49]. Toxicity can also be achieved in a non-susceptible species by feeding with activated toxin [51,52] suggesting that inefficient processing of protoxin precludes toxicity. Fragments of activated toxin produced by intramolecular cleavage can remain toxic when both fragments are present in the gut lumen. For example, Cry4Aa is processed to 45 and 20 kDa fragments from a 130 kDa protoxin [47]. Yamagiwa *et al.* [53] produced GST fusions of both the 45 and 20 kDa fragments. Neither GST (glutathione S-transferase)-45 nor GST-20 was toxic on its own when assayed against *Culex pipiens* larva. However, the presence of both fragments resulted in insecticidal activity [53], indicating that both are needed for toxicity. Activated Cry toxins that exhibit appreciable toxicity against species that lack susceptibility to the protoxin could be modified by insertion of sequences recognized by the proteases of the target insect to facilitate protoxin activation. The following paragraphs discuss the molecular events following processing that may facilitate toxicity.

Toxin binding to insect gut receptors preferentially occurs after appropriate proteolytic cleavage. Greater specific association was noted between processed toxin and brush border membrane vesicles (BBMV) than between protoxin and BBMV [18,48,49,54,55]. Carroll *et al.* [49] found that chymotrypsin treated Cry3A bound specifically to *L. decemlineata* BBMV at the pH of the *L. decemlineata* gut (pH 7.4). In contrast, the Cry3A protoxin did not display specific binding at either the pH found in the insect gut (pH 7.4) or at a pH at which the Cry3A protoxin is soluble (pH 10). Trypsin or chymotrypsin treatment of Cry3Ba and Cry3Ca also resulted in specific binding to the BBMV of *L. decemlineata*, while the protoxin did not exhibit specific binding [49,54,55]. Activated fragments of Cry1Ab following incubation with *M. sexta* gut juices bound BBMV [56]. In ligand blotting experiments, processed Cry4Aa bound BBMV proteins of *C. pipiens* following incubation with gut extracts [56]. These examples demonstrate that processing of the protoxin precedes toxin binding to epithelial cells. These results suggest that following proteolytic cleavage the core toxin is relatively more accessible than protoxin for binding to epithelial cells.

### 4.2. Effects of Activation on Toxin Solubility

Proteolytic activation of toxins can also enhance solubility. Toxins with limited solubility form precipitates that restrict interaction with the insect gut environment and would be expected to have limited toxicity. Cry protoxin solubility is dependent on pH [5,57]. Insect gut pH varies across insect orders; Lepidoptera and Diptera have an alkaline gut pH [5,58], and Coleoptera and Hemiptera have a neutral to acidic gut pH [49,57,59,60]. Limited toxicity against non-susceptible insect species may result from a gut environment that is not conducive to toxin solubilization. However, Cry solubility can change upon proteolytic cleavage. For example, Cry3A protoxin is only soluble under acidic (pH < 4) and alkaline (pH > 10) conditions. The susceptible insect species *L. decemlineata* has a neutral gut pH, an environment where Cry3A protoxin forms insoluble precipitates. However, Carroll *et al.* [49] showed that the solubility of Cry3A changes upon activation with chymotrypsin, increasing its solubility at neutral pH; both Cry3A protoxin and activated toxin exhibit similar toxicity against *L. decemlineata*. These findings suggest that the challenge of toxin insolubility, and the resulting limited toxicity, can be resolved in cases where the insect gut environment is not conducive to solubility.

### 4.3. Activation is Facilitated by Insect Gut Proteases

The examples cited provide compelling evidence that proteolytic cleavage of Cry toxins can be crucial to toxicity. Proper activation of Cry toxins is facilitated by proteases present in the insect gut. Among susceptible species in the orders Lepidoptera and Diptera, the major gut proteases are of the serine type [5,61], while in Coleoptera the major proteases are cysteine and aspartic proteases, although some use cathepsin G serine proteases. Since activation is a crucial step to achieve toxicity, it has been suggested that the type and/or abundance of insect proteases is important in contributing to toxin specificity. As mentioned earlier, Cry toxicity can be achieved in non-target insect species by protoxin activation prior to feeding. Porcar *et al.* [52] demonstrated that a mixture of Cry4A and Cry4B isolated from a recombinant strain of *Bt* subsp. *israelensis* that was trypsin activated displayed enhanced toxicity against the pea aphid (*Acyrthosiphon pisum*) relative to non-activated Cry4, suggesting proteolytic activation as an important limiting step in Cry toxicity against aphids. The major proteases utilized by *A. pisum* are cysteine proteases of the cathepsin L and cathepsin B type [59] whereas dipteran species susceptible to Cry4A and Cry4B have serine proteases [61]. Pea aphids therefore appear to lack the appropriate proteases required for, at least, Cry4 toxin activation.

Insect proteases may also be detrimental to toxicity by degrading or inactivating protoxins by cleavage at inappropriate sites. For example, extensive toxin degradation of several Cry1A toxins is implicated in the insensitivity of *S. exigua*. However, when toxin and protease inhibitors were fed in combination, a synergistic effect was observed against *S. exigua* larva, which was decreased as inhibitor concentrations were reduced [62]. Similarly, increasing the stability of Cry1Fa toxin against *S. frugiperda* larval gut proteases by a cadherin fragment, correlated with increased synergy of toxicity to larvae [63]. In addition to lacking the appropriate proteases required for toxin activation, non-target insects may also harbor toxin degrading proteases [44].

Examples of insufficient protoxin activation and degradation can also be found in cases of field-evolved resistance to *Bt* toxins. Alterations in protease content and/or activity can result in resistance. Cao *et al.* [64] investigated several strains of *H. armigera* with resistance to Cry1Ac and found cases of up-regulated and down-regulated protease activity conferring resistance in strains with low to moderate resistance. Decreased total protease activity resulting in insufficient activation of protoxin was found in some strains, while increased esterase, GST, and chymotrypsin activity resulting in toxin degradation was found in others [64]. These results are supported by *in vitro* work which described a link between insufficient activation of the protoxin and low toxicity [65]. Although protease activity levels were less important in highly resistant strains, modulation of enzyme levels may be important in insects under low selection pressure.

### 4.4. Modification of Cry Protoxin to Facilitate Proteolytic Activation

Our current understanding of how (1) Cry protoxin processing precedes toxicity; (2) insect gut proteases mediate this processing; and (3) insect gut proteases play a role in determining the specificity of Cry toxins by appropriate proteolytic cleavage, suggests a method for broadening the specificity of 3D Cry toxins to include non-target insects as well as combating Cry resistance. Knowledge of the mode of action of the 3D Cry proteins and of insect gut physiology can be combined for the engineering of designer toxins. Walters *et al.* [18] demonstrated the feasibility of this approach by achieving toxicity with modified Cry3A (mCry3A) against the relatively non-susceptible western corn rootworm (*Diabrotica virgifera virgifera*). A chymotrypsin G cleavage site introduced between α-helices 3 and 4 of domain I was cleaved by western corn rootworm gut proteases. The introduced cleavage site resulted in enhanced activity and the activated mCry3A bound specifically to *D.v. virgifera* BBMV [18]. Insertion of protease recognition sequences at appropriate sites should result in enhanced processing of the protoxin to its active form. Similarly, in cases of toxin degradation, toxins can be engineered for removal of deleterious protease sites. Bah *et al.* [50] created Cry1Aa constructs designed to resist degradation in the spruce budworm (*Choristoneura fumiferana*) by mutating potential trypsin and chymotrypsin sites. These modifications resulted in a 2-4 fold increase in toxicity [50]. Understanding of the protease type in the gut of the target insect, as well as the stability and solubility of the candidate Cry toxin will be essential for the success of designer toxins. Fortunately the Cry gene database is vast, and the structure, stability and solubility of many Cry toxins have been categorized. Although Cry3A is the only toxin engineered using modified cleavage sites to date that has resulted in increased toxicity, the success of this approach, as well as the toxicity of activated toxins against non-susceptible insects suggests that the approach can be more widely applied.

### 4.5. Potential Post Binding Modification

The focus of this section has been on the processing events that precede Cry toxin binding. However, post binding cleavage could be important for pore formation, a process referred to as proteolytic nicking [66,67]. This process adds an additional facet to Cry toxin modification by introducing cleavage sites to promote pre-pore structures and pore formation after binding. However, it is unclear how important post binding cleavage is for toxicity. Pore formation may not be dependent on post binding cleavage, and protease activity at the membrane of epithelial cells may inhibit pore formation [68,69,70]. Additional research is needed to clarify post binding events to assess the potential for Cry modification to stimulate pore formation.

## 5. Binding Modifications

Proteolytic activation of Bt toxins, and the subsequent binding to the insect gut epithelium, are important steps for toxicity (reviewed in [4]). Modification of either or both of these steps for a particular Bt toxin should therefore result in altered host range and/or altered toxicity. Several approaches have been taken to modify the binding specificity and affinity of Bt toxins with the ultimate goal of producing designer toxins that target new pest species and counter field evolved resistance. The alteration of Bt toxin binding affinity and specificity can be broken down into four categories: domain or loop swapping between Cry toxins, site-directed mutagenesis, incorporation of binding peptides or fragments from non-Bt toxins, and the generation and subsequent display of Cry toxin mutant libraries on phage.

### 5.1. Domain Swapping

Domain swapping between Cry toxins is perhaps the oldest method used for engineering toxins with novel properties. These large exchanges can also provide information on the function of the exchanged segments. For example, domain III exchanges between Cry toxins have implicated this domain in both toxin binding and host specificity [71,72,73,74]. For this reason, domain III of 3D Cry toxins is used extensively in domain swapping experiments. However, at least one domain II exchange has been successful, with movement of domain II of Cry1Ia to Cry1Ba resulting in a toxin with strong activity against *L. decemlineata* [75].

Exchanging domain III of the 3D Cry1 toxins between Cry1 toxins has been used to enhance toxicity against particular insect pests [71,72,73,75], and to enhance the toxicity of more distantly related 3D Cry toxins [74]. It is often the case that the substitution of all or part of domain III from one Bt toxin to another confers the specificity of the donor toxin upon the recipient toxin [71,72,73]. However, there is an instance of a domain III exchange from a Cry1A-active toxin altering the binding specificity of a coleopteran-active Cry3A toxin. Hybrid Bt toxin, eCry3.1Ab, was generated by the incorporation of a large section of the lepidopteran-active domain III of Cry1Ab into the coleopteran-active modified Cry3A (mCry3A) [18,74] and the resulting hybrid toxin displayed significant activity (approximately 94% mortality) against *D. v. virgifera* [74]. To our knowledge, this is the only documented case of a successful domain exchange between more distantly related Cry toxins.

Although there has been success in using domain swapping as a method for modifying Bt toxins for enhanced efficacy, this approach is limited by a lack of knowledge of what domain II and domain III contribute to the activity of each toxin [71,73]. Until these issues are addressed, the rational design of Bt toxins via domain swapping is hampered.

### 5.2. Site-Directed Mutagenesis

Site-directed mutagenesis of Cry toxins has resulted in impressive enhancements in toxicity against various insect species. Domain II has been heavily targeted for modification as this domain is involved in receptor binding [5]. Modifications have been used to enhance toxicity of Bt toxins that already display activity against the target insect [76], as well as against non-susceptible insects [17,77,78,79]. Loop 1, loop 2, and loop 3 of domain II are of particular interest because they interact with receptors in the gut of the target pest. These loops have been successful targets for alteration, although the success of the loop modifications appears to depend on both the toxin and the target insect. Loop 1 of domain II of Cry3A was altered, resulting in mutants displaying significant increases in toxicity against *L. decemlineata*, the yellow mealworm (*Tenebrio molitor*), and the cottonwood leaf beetle (*Chrysomela scripta*), as well as an increase in binding affinity to *L. decemlineata* BBMV [76]. Modifications to loop 1 and loop 2 of domain II of Cry19Aa made it more Cry4Ba-like: Deletions in loop 2 of domain II and the substitution of Cry4Ba loop 1 residues into loop 1 of Cry19Aa resulted in the mutant toxin, designated 19AL1L2. This toxin showed a 42,000-fold increase in toxicity to the yellow fever mosquito (*Aedes aegypti*) [78]. In stark contrast, when attempting to introduce activity against the southern house mosquito (*Culex quinquefasciatus*) and *C. pipiens* to Cry4Ba, any modifications to loop 1 or loop 2 of domain II resulted in a loss of activity against *A. aegypti* and common malaria mosquito (*Anopheles quadrimaculatus*). However, activity against *C. pipiens* and *C. quinquefasciatus* was successfully introduced to Cry4Ba through modifications to loop 3 of domain II [17]. These results support the variability of Cry toxin interactions with insect gut receptors based on the particular Cry toxin and insect species, an important factor to consider when selecting a toxin to modify for activity against a particular pest. A toxin with a basal level of toxicity against the target pest is optimal for enhancement by toxin modification.

Site-directed mutagenesis was used to dramatically shift the specificity of Cry1Aa, a lepidopteran-active toxin. This toxin was modified for toxicity against *C. pipiens*. Cry1Aa did not cause observable mortality in *C. pipiens* when fed at a concentration of 100 μg/mL. Loop 1 and loop 2 of domain II were modified to mimic loop 1 and loop 2 of Cry4Ba in both amino acid sequence and loop length. The resulting mutant toxin lost activity against the lepidopteran *M. sexta* but gained activity against *C. pipiens*, with an LC_50_ of 45 μg/mL [79], marking a considerable improvement in toxicity against this insect. This work further supported the importance not only of the amino acid residues involved in binding to the receptors, but of the length and shape of the loops. Loop 1 of Cry1Aa is shorter than that of Cry4Ba, while loop 2 of Cry1Aa is longer than loop 2 of Cry4Ba. When mutant toxins were produced with a longer loop 2, mosquitocidal activity was not observed [79].

Site directed mutagenesis has been used successfully to modify Bt, and offers a powerful tool to combat resistance in Bt susceptible pest species, as well as to broaden the spectrum of insect pests targeted by Bt toxins. In addition, this approach has provided important insight into the specific Cry toxin residues that interact with insect gut receptors.

### 5.3. Use of Synthetic Peptides and Other Non-Bt Toxin Regions

The use of synthetic gut binding peptides and non-Bt toxin fragments in the engineering of Bt toxins is a relatively new approach, which has the potential to easily enhance the toxicity of Bt toxins against pests which have little to no susceptibility. The first example involved the fusion of the non-toxic lectin, ricin B-chain, to the *C*-terminus of lepidopteran-active Cry1Ac via a four amino acid linker region, to produce a toxin designated BtRB [80]. BtRB was expressed in rice and maize and subsequently tested against the striped stem borer (*Chilo suppressalis*), cotton leaf worm *S. littoralis*, and the maize leafhopper (*Cicadulina mbila*). Mortality was increased against the Cry1Ac susceptible *C. suppressalis*, from approximately 17% to 75% on maize and from 20%–30% to 60%–90% on rice. BtRB substantially increased the mortality of Cry1Ac-tolerant *S. littoralis*, from less than 20% on control maize plants to 78% on BtRB-expressing maize plants at 4 days. The mortality of *C. mbila* fed on control maize plants was about 20%, while the mortality on BtRB-expressing maize plants was about 95%. Although the ricin B-chain did enhance the activity of Cry1Ac, it did not do so indiscriminately as neither Cry1Ac nor BtRB displayed activity against the bird cherry-oat aphid (*Rhopalosiphum padi*) [80]. This result may reflect the specificity of ricin B binding to galactose residues and the abundance of these residues in the insect gut, however as discussed above, a number of other factors affect Cry host range. The introduction of toxicity to a Bt toxin against a hemipteran, *C. mbila*, was significant however, as few Bt toxins display toxicity against hemipteran pests [52,81,82]. In a similar study, replacing domain III of Cry1Ac with *Allium sativum* agglutinin (ASAL; a mannose-binding lectin) resulted in a 30-fold increase in toxicity against *H. armigera* and an 8-fold increase against *P. gossypiella*, as well as an expanded binding profile to *H. armigera* BBMV on a ligand blot [83].

Through the addition of lectins to Cry1Ac, toxicity against susceptible and non-susceptible insect pests was enhanced, albeit in an unpredictable manner. The incorporation of short gut binding peptides, identified via a phage display library, into Bt toxins provides a method to target specific pests that lack susceptibility to Bt toxins. To date, two papers have been published on the use of receptor binding peptides to modify Bt toxins.

**Figure 2:**
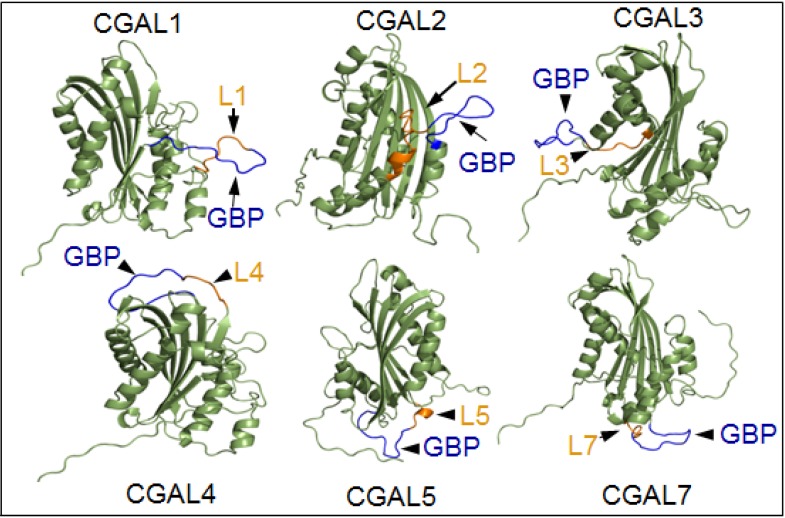
Models for addition mutants of Cyt2Aa (CGALn) with the pea aphid gut binding peptide GBP3.1 (GBP) incorporated into various loops (Ln) and exposed on the surface of Cyt2Aa. Homology based models were developed by LOMETS. Source: [85]. Of these modified toxins, the binding and toxicity of CGAL1, CGAL3 and CGAL4 in the pea aphid were both increased. Engineering to produce CGAL2, CGAL5 and CGAL7 resulted in loss of Cyt2Aa toxicity to mosquito larvae [81].

A 12-aa *A. pisum* gut binding peptide, designated GBP3.1, previously identified by feeding *A. pisum* on a phage display library [84] and subsequently found to bind to *A. pisum* alanyl aminopeptidase-N (APN), was incorporated into different loops of the cytolytic Bt toxin Cyt2Aa through the addition or substitution of amino acids (Figure 2) [85]. The incorporation of GBP3.1 into loops 1, 3 and 4 of Cyt2Aa resulted in mutants with significantly higher binding to *A. pisum* BBMV, as measured by pull-down assays and surface plasmon resonance. The increase in binding was accompanied by a significant improvement in toxicity to *A. pisum*, lowering the LC_50_ from >150 μg/mL to 10–29 μg/mL. The mutant Cyt2Aa toxins also displayed improved activity against the green peach aphid (*Myzus persicae*), lowering the LC_50_ from >150 μg/mL to 43–93 μg/mL [85].

Interestingly, while GBP3.1 bound to APN, a glycosylphosphatidyl inositol (GPI)-anchored membrane bound protein that has been implicated as a receptor for Bt toxins, in the case of Cyt and possibly the 3D Cry toxins, the specific protein which binds the toxin may not matter.

The “sequential binding model” of 3D Cry toxin-receptor interactions, where Cry toxins interact with a number of insect midgut proteins that facilitate formation of oligomeric structures and membrane insertion [86], supports the concept that receptor identity may not be as fundamental as once thought. For example, the highly toxic Cry11Ba toxin binds at least four different GPI-anchored proteins (alkaline phosphatase, aminopeptidase, and two glucosidases), plus two cadherin-like proteins in the midgut of the malarial mosquito *Anopheles gambiae* [87,88]. It may be advantageous from the perspective of insecticidal potency to have multiple midgut binding proteins acting as “accessory” receptors to gather toxin at the midgut brush border protein level. The concept of increasing toxin retention leading to increased potency is suggested by the following example: an Asian longhorn beetle (*Anoplophora glabripennis* Motschulsky) Cx-cellulase binding peptide, designated PCx, was used to modify Cry3Aa. PCx was fused to either the *N*-terminus (PCx-Cry3Aa) or *C*-terminus (Cry3Aa-PCx) of Cry3Aa. PCx-Cry3Aa and Cry3Aa-PCx both showed increased toxicity against the Mulberry longhorn beetle (*Apriona germari* Hope), roughly doubling the mortality of *A. germari* compared to Cry3Aa. In addition the retention of PCx-Cry3Aa in the midgut of *A. germari* was significantly increased relative to Cry3Aa [89]. This approach suggests that increasing the “bindability” of Cry toxins may augment recognized Bt toxin receptors such as cadherin, APN, and ALP. Alternatively, increased retention of Bt toxin in the insect gut, as well as proximity to the gut epithelium, may in itself be sufficient for increasing toxicity and target range.

### 5.4. Phage Display

A phage display library expresses a diverse array of peptides or proteins on the surface of bacteriophage. Peptides or proteins that have a higher target binding affinity are selected by screening the phage display library against purified receptor or BBMV immobilized on a substrate [36,90,91,92]. Alternatively, feeding assays can be used to isolate peptides or proteins that bind to the gut epithelium of a targeted insect [84]. Unbound phage are removed by washing. Bound phage are then eluted and amplified in *E. coli* for subsequent rounds of selection, and selected phage sequenced to identify sequences encoding peptides of interest.

Phage display libraries expressing mutant Cry toxins offer a potentially powerful method for engineering Cry toxins. The most important aspect of this methodology is the successful display of complete Cry toxins on the surface of a phage, followed by the generation of phage display libraries expressing mutant Cry toxins and the subsequent selection of mutant Cry toxins via binding affinity to BBMVs or purified gut receptors.

The display of Cry toxins on the surface of phage has been attempted several times over the past twenty years, with initial attempts resulting only in partial success. The earliest published attempt to display a Cry toxin on a phage succeeded in only displaying domain II or only loop 2 of domain II of Cry1Aa on M13 phage, despite attempts to display the activated toxin. In this case, the *E. coli* were unable to produce the activated toxin, possibly due to Cry1Aa being directly or indirectly toxic to the cells [93]. Despite not displaying activated Cry1Aa, the successful display of domain II and loop 2 of domain II still allowed for the generation of combinatorial libraries that could be used to identify sequences with higher binding, and thus potentially enhanced toxicity, once incorporated into the complete toxin. This goal could also be accomplished by incorporating short binding peptide sequences identified via phage display library into any number of Bt toxins [84,85,86,87,88,89]. A concurrent attempt to display Cry1Ac on the surface of a phage was more successful in that Cry1Ac was displayed, and was active against *M. sexta* and *H. virescens*. However, when exposed to BBMV, Cry1Ac was cleaved from the phage, limiting *in vivo* and *in vitro* applications [94].

Recent efforts with phage display of Bt toxins have been more successful. Cry1Ac was successfully displayed on λ phage by fusing the toxin to the structural D protein, an essential protein for phage stability found in the head of λ phage. The fusion protein successfully bound to brush border membrane proteins, purified from a preparation of *M. sexta* BBMV. However, the display of Cry1Ac on λ phage did result in a 20-fold decrease in toxicity against *M. sexta* [95]. The decrease in toxicity may have resulted from the λ phage inhibiting secondary binding events or pore formation. Fusion of Cry1Ac to the T7 phage structural gene 10B provided comparable mortality in *M. sexta* to Cry1Ac [96]. Despite the decreased toxicity seen in some instances, the successful display of Cry1Ac on the surface of λ phage and interaction of the displayed toxin with gut receptors is a significant step forward for use of phage display libraries for selection of toxins with enhanced binding properties. The screening of libraries displaying complete toxins under *in vivo* conditions is less practical, as toxins with increased binding are expected to damage the gut epithelium or kill the insect, thereby hampering isolation of bound phage for further enrichment.

There have been a few attempts to generate and screen Cry mutant libraries for enhanced binding and toxicity via phage display [36,90,91,92]. Two of these attempts involved the generation of domain II mutant libraries [90,92], while the other two mutant libraries were generated by introducing mutations into random locations in Cry genes [36,91]. However, a noted difficulty with this approach is generation of inclusive mutant libraries [92]. This problem could be alleviated by targeting shorter amino acid sequences for mutation [90], decreasing the number of possible permutations of the target sequence, thereby decreasing the number of mutants needed for an inclusive library. As stated previously, an important consideration is the probable difficulty of *in vivo* biopanning, as toxin-induced mortality could hamper elution of bound phage from the insect gut. This limitation restricts library screening to the use of purified receptors [90,92], or BBMV [36,91], which do not necessarily represent biologically relevant conditions, and could lead to the selection of toxins that are not active under *in vivo* conditions.

The generation of mutant Cry toxin libraries and their display on various phage provides a powerful approach for the selection of toxins with enhanced efficacy against target insect pests, but with important limitations. Production of a representative mutant library, particularly when altering specific regions of a Cry toxin, is an important but technically limiting step. Phage display library screens conducted *in vitro* may not result in toxins active under *in vivo* conditions.

**Table 1:** Selected modifications made to Bt toxins for improved efficacy.

Type of Modification	Bt Toxin	Insect Targeted	Reference
**Truncation**			
Truncation and selection of mutant toxins from phage display library based on binding affinity	Cry1Ia	*Telchin licus licus*	[36]
Truncated Helix α-1 of Domain I	Cry1A	*Pectinophora gossypiella*	[39]
Truncated Helix α-1 of Domain I	Cry1A	*Plutella xylostella*; *Ostrinia nubilalis*	[40]
Truncated *N*- and *C*-terminus	Cry1 HD-1 Dipel	*Manduca sexta*	[27]
Truncated *C*-terminus	Cry1 HD-1 Dipel	*Manduca sexta*	[37]
Truncated *C*-terminus	Cry1Ba	*Leptinotarsa decemlineata*	[35]
Truncated *C*-terminus	Cry1C	*Spodoptera exigua*	[43]
Truncated *N*-terminus	Cry2A	*Spodoptera littoralis*; *Helicoverpa armigera*; *Agrotis ipsilon*	[16]
Truncated *C*-terminus	Cry4A	*Culex pipiens*	[42]
Truncated *N*-terminus	Cry11	*Spodoptera frugiperda*; *Trichoplusia ni*	[32]
Truncated *N*- and *C*-terminus	Cry11A	*Aedes aegypti*	[31]
Truncated *C*-terminus	Cry15A	*Cydia pomonella*	[34]
Truncated *N*- and *C*-terminus	Cry1A	*Pieris brassicae*; *Manduca sexta*	[26]
**Activation**			
Chymotrypsin/cathepsin G cleavage site in domain I	Cry3Aa	*Diabrotica virgifera virgifera*	[18]
Mutation of potential trypsin and chymotrypsin sites to resist degradation	Cry1Aa	*Choristoneura fumiferana*	[50]
**Domain swapping**			
Domain III swap with Cry1Ab	mCry3Aa	*Diabrotica virgifera virgifera*	[74]
Domain III swap with Cry1C	Cry1Ab	*Spodoptera exigua*	[72]
Domain III swap with Cry1Ca	Cry1Ab Cry1Ac Cry1Ba Cry1Ea Cry1Fa	*Spodoptera exigua*	[71]
Domain III swap with Cry1Ac	Cry1Ca Cry1Fb Cry1Ba Cry1Da Cry1Ea	*Heliothis virescens*	[73]
Domain II and domain III swapping between Cry1Ia and Cry1Ba	Cry1Ia Cry1Ba	*Leptinotarsa decemlineata*	[75]
**Site-directed mutagenesis**			
Substitutions in loop 1, loop 2, and loop 3 of domain II	Cry4Ba	*Culex pipiens*; *Culex quinquefasciatus*	[17]
Substitutions in loop 1 and loop 2 of domain II	Cry19Aa	*Aedes aegypti*	[78]
Subsitutions/deletions in domain II	Cry1Ab	*Lymantria dispar*	[77]
Substitutions/deletions in loop 1 and loop 2 of domain II	Cry1Aa	*Culex pipiens*	[79]
Substitutions/deletions in loop 1 of domain II	Cry3A	*Tenebrio molitor*; *Leptinotarsa decemlineata*; *Chrysomela scripta*	[76]
**Peptide addition**			
Incorporation of binding peptide into various loops	Cyt2Aa	*Acyrthosiphon pisum*; *Myzus persicae*	[85]
*N*-terminal fusion of ricin B-chain	Cry1Ac	*Chilo suppressalis*; *Spodoptera littoralis*; *Rhopalosiphum padi*; *Cicadulina mbila*	[80]
Replacement of domain III with Allium sativum lectin	Cry1Ac	*Helicoverpa armigera*; *Pectinophora gossypiella*	[83]
*N*- and *C*-terminal fusion of binding peptide	Cry3Aa	*Apriona germari* Hope	[89]
**Phage Display**			
Selection of mutant toxins from phage display library based on binding affinity	Cry1Aa	*Bombyx mori*	[90]
Selection of mutant toxins from phage display library based on binding affinity	Cry8Ka	*Anthonomus grandis*	[91]
Selection of mutant toxins from phage display library based on binding affinity	Cry1Aa	*Bombyx mori*	[92]

## 6. Concluding Remarks

Toxins derived from the bacterium *B. thuringiensis* have been used extensively to manage insect pest populations, traditionally through foliar sprays and more recently via transgenic crops. A thorough understanding of the mode of action of these toxins is required for full exploitation of their potential. Some insects of agricultural and human health importance lack susceptibility to Cry toxins while other species evolve resistance in response to intense selection pressure. The lack of toxicity in these cases likely involves interference with crucial steps in toxin action: *N*- or *C*-terminal cleavage, intramolecular proteolytic cleavage, and/or binding to the insect gut epithelium (Figure 1). Based on understanding of Cry toxin mode of action and the gut physiology of targeted pests, specific modifications have been made to counter limitations to toxicity. Toxin modification has resulted in toxins with increased efficacy against various target pests (Table 1), and increased understanding of toxin mode of action. Incorporation of such modified toxins into current management strategies will maximize the use of Bt as a tool for crop protection and for management of insect borne disease.
